# Effects of Religious Practice and Teachings about Sexual Behavior on Intent to Vaccinate against Human Papillomavirus

**DOI:** 10.3390/vaccines10030397

**Published:** 2022-03-04

**Authors:** David S. Redd, Jamie L. Jensen, Savannah J. Hughes, Kendall Pogue, Chantel D. Sloan-Aagard, Dashiell S. Miner, Jessica D. Altman, Triston B. Crook, Lydia Zentz, Ruth J. Bodily, Brian D. Poole

**Affiliations:** 1 Department of Microbiology and Molecular Biology, Brigham Young University, Provo, UT 84602, USA; dredd2@byu.edu (D.S.R.); sav002@byu.edu (S.J.H.); kpogue2@byu.edu (K.P.); dminer3@byu.edu (D.S.M.); jaltman4@byu.edu (J.D.A.); tc499@byu.edu (T.B.C.); ldzentz@byu.edu (L.Z.); ruthjb@byu.edu (R.J.B.); 2 Department of Biology, Brigham Young University, Provo, UT 84602, USA; jamie.jensen@byu.edu; 3 Department of Public Health, Brigham Young University, Provo, UT 84602, USA; chantel.sloan@byu.edu

**Keywords:** human papillomavirus, sexually transmitted infection, vaccine attitudes, vaccine hesitancy, Christian religious views

## Abstract

Human papillomavirus (HPV) is the most common sexually transmitted infection in the United States. Most infections are mild and clear without treatment in 1 to 2 years. Some HPV strains result in persistent infection, which can cause various cancers, including cervical, penile, anal, mouth, and throat cancers. Vaccines have been developed that provide protection against the highest risk HPV strains. Despite HPV vaccines having been proven to be safe and effective, uptake has been low. Religiosity has been negatively correlated with HPV vaccine uptake in some studies. It is hypothesized that religiosity and Christian religious affiliation could impact parents’ decision to vaccinate their children against HPV via teachings and beliefs about sexual behaviors. A survey was distributed to participants to determine what factors, including religiosity and views about sex, impacted HPV vaccination. The survey results (*n* = 442) were analyzed using confirmatory factor analysis, structural equation modeling, and univariate factor analysis. The association between religious practice and vaccine attitudes were complex, with religious practice slightly positively correlated with pro-vaccine attitudes and vaccine knowledge, but also with the belief that religious adherence to expectations surrounding sexual behavior will protect children from HPV infection, as well as more negative views towards vaccines, in general.

## 1. Introduction

Human papillomaviruses (HPV) are a family of human, non-enveloped, double-stranded DNA viruses [1]. HPV is the most commonly sexually-transmitted infection in the United States. It is estimated that over 80% of sexually active individuals will contract HPV sometime during their lives [1,2]. HPV is generally transmitted through skin-to-skin or sexual contact, where it infects cutaneous and mucosal epithelium [2,3]. Most HPV infections do not cause serious symptoms and resolve without treatment within 1 to 2 years [2]. Because HPV often presents asymptomatically, it can be passed unknowingly between sexual partners. Although many HPV strains are not a serious concern, some strains can cause persistent infection, which can result in genital warts and cancer in mucosal membranes, including cervical, anal, penile, and throat cancers. HPV is the primary causative agent of cervical cancer; HPV is responsible for over 95% of cervical cancer cases. Oncogenic HPV strains are classified as high risk; strains 16 and 18 are the most dangerous, causing 70% of HPV-associated cancers [3]. 

Due to the risk presented by persistent HPV infection and the cancers associated with it, significant effort was made to develop a vaccine. Three vaccines have been approved by the Food and Drug Administration (FDA) for use in the United States. Multiple studies have determined that the three approved vaccines have an acceptable safety profile and are effective at preventing high-risk HPV infection [4]. HPV vaccination is recommended for both males and females, ages 9 to 45 [5,6]. HPV vaccines provide the best protection if administered before an individual becomes sexually active. It is recommended that vaccination be administered during the early teens, but it can be administered later [5,7]. Vaccination efforts have been highly effective at reducing incidence and transmission of strains covered by the vaccine [8]. It is anticipated that cervical cancer could be completely eliminated in areas with high rates of vaccine uptake [9]. 

Despite HPV vaccines having proven effectiveness and an acceptable safety profile, the vaccination rate in the United States is low. Recent estimates of adolescent (ages 11–17) vaccination coverage show that 41.9% of females and 28.1% of males have completed a vaccination series [10]. The reported vaccination rates for young adults are even lower than adolescents. The US department of Health and Human Services (HHS) estimates that, in the United States, less than half of young adults (ages 18–26) have received an HPV vaccine dose, and only 22% have completed a vaccine series [11]. HPV vaccination rates are increasing in teenagers but still fall below the vaccination target of 80%. HPV vaccination rates are also well below the rates of other vaccines recommended for adolescents, such as Tdap and MenACWY [10,11]. This indicates that the HPV vaccine is not being routinely recommended or administered when other adolescent vaccines are administered. HPV vaccination rates of adolescents are closely monitored and studied; however, there are less data on young adult vaccination rates. A study looking at vaccination trends in the 2010–2018 National health interview survey found that participants who reported having at least one dose increased between 2010 and 2018, from 32 to 55% for females and 2 to 34% for males. The study also found that 4% of females and 3% of males initiated vaccination between ages 18 and 21. In comparison, 68.1% of adolescents have received one or more doses [12]. Young adults have more control over health decisions than adolescents but may have less access to health care services, be unaware they did not receive the vaccine, or not actively seek medical care because they believe themselves to be healthy. If the HPV vaccination series is not initiated as an adolescent, the series is less likely to be completed [12]. There are many factors that could impact HPV vaccine uptake, including access to vaccination, health care provider recommendations, parental attitudes, religiosity, risk of infection, vaccine mandates, or sexual activity.

Multiple studies have shown a negative correlation between religious affiliation and HPV vaccine uptake [13,14,15]. In a study of female college students, the impact of religiosity/spirituality on sexual decision making was assessed. Bivariate analysis showed that sexual activity and religious/spiritual beliefs were independently associated with HPV vaccine uptake. However, only sexual activity was significantly associated with vaccination in this study. After correcting for socio-demographic variables, sexual activity was found to fully explain the relationship between religious/spiritual beliefs and HPV vaccination [13]. This could indicate that the influence of religiosity on sexual behavior could impact HPV vaccine uptake. A national study investigated factors that influence HPV vaccination initiation. Survey participants who were sexually active and participated in religious services less than once a month were more likely to report initiation of HPV vaccination [14]. Another study of young adults in Utah found that participants who belonged to an organized religion were significantly less likely to have received a provider recommendation and initiated or completed an HPV vaccination series [15]. Although these studies suggest that religiosity can have a negative impact on HPV vaccine uptake, more research in this area is necessary.

Understanding the impact of religiosity, religious affiliation, and religious beliefs about sexual behavior on whether parents decide to vaccinate their children is important, because HPV vaccination is recommended for children in their early teens, before the initiation of sexual activity. In a survey study of parents and caregivers of daughters, it was found that parents who frequently attended religious services were more likely to decline vaccination than their less-religious peers [16]. In a focus group study of rural parents, the impact of spirituality and religiosity on HPV vaccination attitudes was investigated. It was found that religiosity and spirituality influence health choices and play an integral role in the parents’ life. The study also showed that parents in rural communities have restricted access to healthcare providers; therefore, the religious community could play a valuable role in encouraging parents to vaccinate their children [17]. The previous studies show that the impact of religiosity and religious affiliation is complex; religiosity can negatively impact parents’ decision to vaccinate children against HPV, but addressing religious concerns could also be an avenue to increasing vaccine acceptance. We intend to explore the complex relationships found in these prior studies using structural equation modeling, in order to look at how variables influence each other, in terms of intent to vaccinate against HPV. The focus of this research is not on teachings about vaccination in church, which are likely minimal, but on how factors associated with religiosity affect attitudes towards vaccines. Since attitudes towards vaccines by the public can have a strong effect on public policy, such as vaccine mandates to attend public school, efforts to improve vaccine attitudes in this population may have far-reaching effects. 

The aim of this study was to determine how Christian religious activity and teachings about sexual relationships affects willingness to vaccinate children against HPV. We hypothesized that increased religious practice and stronger views about sexual relationships being sinful would affect such factors as trust in medicine, attitudes towards vaccines in general, belief that lifestyle protects against infection, and knowledge about vaccines and HPV. We further hypothesized that these factors would influence intent to vaccinate children against HPV. By understanding how these factors relate to each other, we hope to identify areas that can be emphasized in public health messaging or other mechanisms to improve vaccine uptake in this population. 

## 2. Materials and Methods

### 2.1. Survey of Christian Parents

Parents were invited to participate in a cross-sectional study by completing an online survey designed to assess attitudes toward HPV vaccination, as well as Christian religious views and affiliation. The survey was distributed electronically by Qualtrics (Provo UT), using their nationwide survey panel. Inclusion criteria included self-identification as Christian and being the parent of at least one child under the age of 11. Education level of the respondents was also used to determine participation, to ensure conformity with the education proportions in the United States, as a reference population, and diminish sampling bias. For an optimal structural equation model, 442 complete responses were recorded. Structural equation modeling is recommended to have at least 20 respondents per factor [18]. With our nine factors, we need at least 180 respondents, so our sample size is more than adequate. Furthermore, post-hoc analysis, using confirmatory factor analysis, is effective at evaluating sample size [19], and our confirmatory factory analysis showed excellent validity with our sample. Incomplete responses were not provided by the surveying company. Quality control was performed using a timing method, whereby any participant who spent less than half the mean time completing the survey was rejected. The survey was open from 9 April 2021 to 20 May 2021.

### 2.2. Survey Description

The survey consisted of 10 sections. The first section was an informed consent page, which included a short explanation of the survey and stated that attitudes toward the human papillomavirus vaccine were being studied. Participants were told that participation was optional, the survey would take approximately 20 min to complete, survey responses would be used for research purposes, and all responses would be kept anonymous. Respondents who were willing to participate in the survey could accept the terms and conditions and continue with the survey. Respondents who did not accept the conditions were thanked for their time, and the survey concluded. The study was carried out under the principles of the Declaration of Helsinki. The study received ethical approval from the institutional review board of Brigham Young University (Protocol #E2021-052).

The second section of the survey assessed demographic information, including religious affiliation, number of children, age, sex, race, education, political affiliation, and socioeconomic status. The third section assessed participant views on the connection between sexual inactivity, due to religious beliefs, and contracting HPV (*Beliefs that Religious Adherence Protects Against HPV*). The fourth section assessed views towards vaccines, in general (*Positive Attitudes Toward Vaccines*), and the HPV vaccine, in particular (*Fear of HPV Vaccine Side-effects* and *Intent to Vaccinate*). The fifth survey section had questions evaluating participants’ knowledge about, and understanding of, vaccines (*Vaccine Knowledge*) and HPV (*HPV Knowledge*). The sixth section assessed participants’ religiosity (*Religious Practice*, *Religious Influence*, and *Religious Hope*). The seventh section assessed how participants’ religious affiliation viewed vaccines *(Pro-Vaccine Religious Views*). The eighth section assessed how religion influences participants’ views on sexual behavior (*Religious Encouragement of Premarital Abstinence*). The ninth section assessed the parental/peer influence on sexual behavior (*Parental/Peer Influence on Sexual Behavior*). The final section of the survey assessed participants’ trust in modern medicine (*Trust in Modern Medicine*). The survey itself was checked for face validity by a virologist (Dr. Poole), specialist in biological education and religious influences (Dr. Jensen), and public health expert (Dr. Sloan-Aagard). Intelligibility was checked by at least two undergraduate students (the full survey can be found in the Supplementary Materials Table S1).

### 2.3. Confirmatory Factor Analysis and Structural Equation Modeling

To validate our survey, we used confirmatory factor analysis (CFA) to confirm that the questions included in our surveys accurately represented our latent variables; to test the relationships between latent variables, we performed structural equation modeling (SEM). Before starting analyses, we cleaned and organized the data using SPSS statistics software (IBM 2021 Armonk, NY, USA). Mplus software, ver. 8 (Muthen and Muthen, 1998–2010, Los Angeles, CA, USA), was used to perform both CFA on the measurement portion and SEM on the structural portion of our models. Each latent variable in the model was represented by three or more survey items. CFA was performed with a request for modification indices. Items were removed until fit indices (root mean square error approximation (RMSEA), comparative fit index (CFI), Tucker–Lewis index (TLI), and standardized root mean square residual (SRMR)) were acceptable. Instruments were combined into a full measurement model to ensure fit before commencing structural modeling. SEM was performed on two hypothetical models, comprised of validated latent variables and income as a covariate in Model A. 

### 2.4. Univariate Analysis

Univariate analyses were performed using Pearson’s correlation analysis. The *intent to vaccinate* score was derived by combining the items from Section 4 of the survey. A score for *belief in vaccine efficacy* was generated by combining the scores for the questions “Vaccines are more helpful than harmful” and “vaccines are effective at preventing disease.” A score for *vaccine safety* was generated by combing the responses to “Vaccines contain dangerous toxins” and “Vaccines often have severe side effects.” These were then compared to the *intent to vaccinate* score using Pearson correlation. A score for *general vaccine knowledge* was generated by scoring the responses to the questions “Smallpox has been eliminated because of mass vaccination,” “Vaccines increase the risk for allergies,” “Unvaccinated children are more resistant to infections,” “Routine immunizations can be given while a child is on antibiotics for an ear infection,” “Current scientific evidence supports associations between vaccines and chronic conditions such as autism or multiple sclerosis”, and “The Food and Drug Administration (FDA) approval process for vaccines is the same as that for other drugs and pharmaceuticals.” These scores were compared to the *intent to vaccinate* score, using Pearson correlation.

## 3. Results

### 3.1. Characteristics of Study Respondents

We began analysis of the survey data by summarizing the baseline characteristics of the study respondents (Table 1). The majority of respondents were between ages 26 and 45 (77.59%). Approximately three-fifths (60.4%) of respondents identified as female, and two-fifths identified as male (39.60%); none of the respondents identified as non-binary or third gender. Most respondents identified as partnered (75.11%). Approximately half of the respondents have two children (49.77%). The respondents were fairly well-educated with over half (53.4%) having completed at least an associate’s degree. Income was relatively evenly distributed. The three most selected religious affiliation were Christian (non-denominational) (38.91%), Catholic (30.77%), and Baptist (13.12%).

### 3.2. Confirmatory Factor Analysis

**Figure 1:**
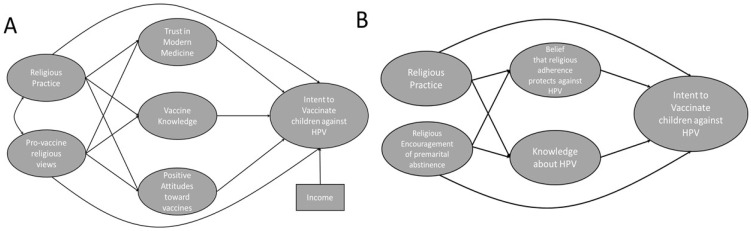
Hypothetical models, illustrating components of hypotheses. (**A**) We hypothesize that religious practice and pro-vaccine religious views influence trust in modern medicine, vaccine knowledge, and general positive attitudes toward vaccines, which, in turn, influence intent to vaccinate children against HPV. (**B**) We hypothesize that religious practice and encouragement of premarital abstinence influence beliefs that religious adherence protects against HPV and knowledge about HPV, which, in turn, influence intent to vaccinate. These connections are illustrated visually in the models.

Confirmatory factor analysis showed that each latent variable fit the data well. CFA models were run for each structural equation model (see Figure 1A,B), the remaining latent factors used for univariate analyses and combined model. Two items were removed from the latent variable, *positive attitudes toward vaccines*, due to lack of fit (“Vaccines often have severe side effects” and “Vaccines contain dangerous toxins”). The survey section on attitudes toward the HPV vaccine was divided into two latent variables: *fear of HPV vaccine side-effects*, which consisted of items 1, 4, and 5; and *intent to vaccinate*, which consisted of items 2, 3, 6, and 7. One item was removed from the latent variable, *vaccine knowledge*, due to lack of fit (“Smallpox has been eliminated because of mass vaccination”). Two items were removed from the latent variable, *HPV knowledge*, due to lack of fit (“Only a small minority of people will catch HPV during their lives” and “HPV causes cancer in women but not men”). Additionally, one item was removed from the latent variable, *trust in modern medicine*, due to lack of fit (“Doctors sometimes do not pay attention to or disregard what their patients are telling them”). Fit statistics are shown in Table 2. CFA models are included in the Supplementary Materials (Figures S1 and S2).

### 3.3. Structural Equation Modeling

SEM on Model A shows a robust fit, as indicated by fit statistics and probability scores (see Table 3). The model indicates that respondents with higher religious practice have a slightly higher intent to vaccinate their children against HPV (+0.158). Respondents with higher religious practice also had higher vaccine knowledge (+0.639). Vaccine knowledge is not a significant predictor of intent to vaccinate. Respondents with higher religious practice have a more negative attitude to vaccines, in general (−0.358). Respondents who view vaccines positively have a higher intent to vaccinate their children (+0.590). Lower attitudes toward vaccination negatively impacts intent to vaccinate. Respondents who indicated that their religion views vaccines positively have more trust in modern medicine (+0.615), less vaccine knowledge (−0.245), and higher attitudes towards vaccines in general (+0.828). There is not a significant relationship between trust in modern medicine and intent to vaccinate. There is also not a significant relationship between vaccine knowledge and intent to vaccinate. Income positively influences intent to vaccinate (+0.157). 

**Figure 2:**
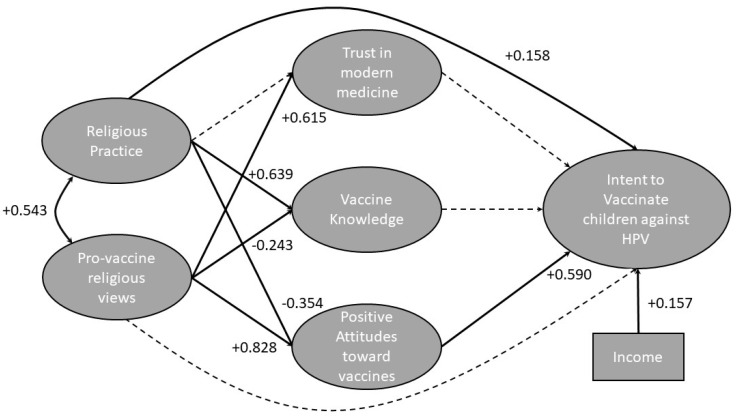
Results of structural equation model A. We hypothesize that religious practice and pro-vaccine religious views affect trust in modern medicine, vaccine knowledge and positive attitudes toward vaccines, which in turn affects parents’ intent to vaccinate against HPV. Bolded lines indicate the relationship is significant, the numbers adjacent to the lines indicate the strength and direction of the relationship. The biggest influence on intent to vaccinate is positive attitudes toward vaccines (+0.590), which is negatively influenced by religious practice (−0.354) and positively influenced by the views of respondent’s religion toward vaccines.

In Model A (Figure 2), a positive attitude toward vaccines, in general, is the strongest predictor of intent to vaccinate; religious practice negatively impacts vaccine attitudes, whereas positive religious views on vaccines positively impacts vaccine attitudes. 

**Figure 3:**
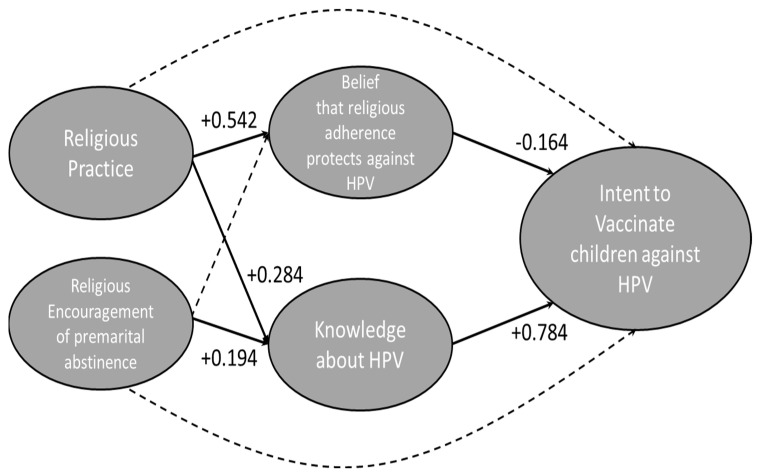
Structural Equation modeling results. Model B, representing the second component of our hypothesis. We hypothesize that religious practice and religious encouragement of premarital abstinence affects the belief that religious adherence protects against HPV and knowledge about HPV, which, in turn, both affect intent to vaccinate against HPV. Bolded lines indicate that a relationship is significant; the numbers adjacent to the lines indicate the strength and direction of the relationship.

SEM on Model B also shows a robust fit, as indicated by fit statistics and probability scores (Table 2). The model (Figure 3) indicates that religious practice is a significant predictor of a belief that religious adherence protects against HPV (+0.542). This belief, in turn, negatively impacts intent to vaccinate (−0.164). Respondents with high religious practice have higher knowledge about HPV (+0.284), which positively impacts intent to vaccinate (+0.784). Respondents whose religion highly emphasizes abstaining from sex before marriage have slightly higher knowledge of HPV (+0.194), which positively influences intent to vaccinate (+0.784). Neither religious practice nor religious encouragement of premarital abstinence has a direct impact on intent to vaccinate. Knowledge about HPV is the strongest predictor of intent to vaccinate; both religious practice and encouragement of abstinence before marriage positively affect knowledge about HPV. 

### 3.4. Univariate Factor Analysis

**Figure 4:**
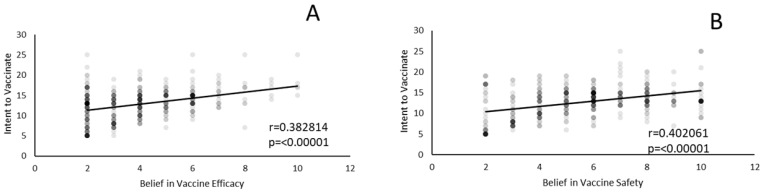
Intent to vaccinate correlates with views of safety and efficacy: There is a strong correlation between an individual’s intent to vaccinate their children against HPV and beliefs in the safety and efficacy of HPV vaccines. The y-axis indicates intent to vaccinate, and the x-axis indicates beliefs in the safety and efficacy of the vaccine. (**A**) Individuals who believe that the vaccine is effective have a higher intent to vaccinate their children (r = 0.3828, *p* ≤ 0.00001). (**B**) Individuals who believe that the vaccine is safe have a higher intent to vaccinate their children (r = 0.4021, *p* ≤ 0.00001).

Univariate correlation analysis was preformed to determine whether belief in vaccine efficacy and safety impact respondents’ intent to vaccinate their children against HPV (Figure 4). There is a strong positive correlation between intent to vaccinate and belief in vaccine efficacy (r = 0.3828, *p* < 0.00001). The magnitude of this effect was a change in intent to vaccinate score of 5. These scores rose from a low median of 12 to a high of 17, with increasing belief in vaccine efficacy. Univariate analysis also indicated that there was a strong positive correlation between views on the safety of the HPV vaccine and intent to vaccinate (r = −0.3828, *p* ≤ 0.00001). Intent scores rose from a low of 6 to a high of 14, with increasing confidence in vaccine safety. For both efficacy and safety scores, there was a plateau effect, with approximately the top third of scores having the same median values.

**Figure 5:**
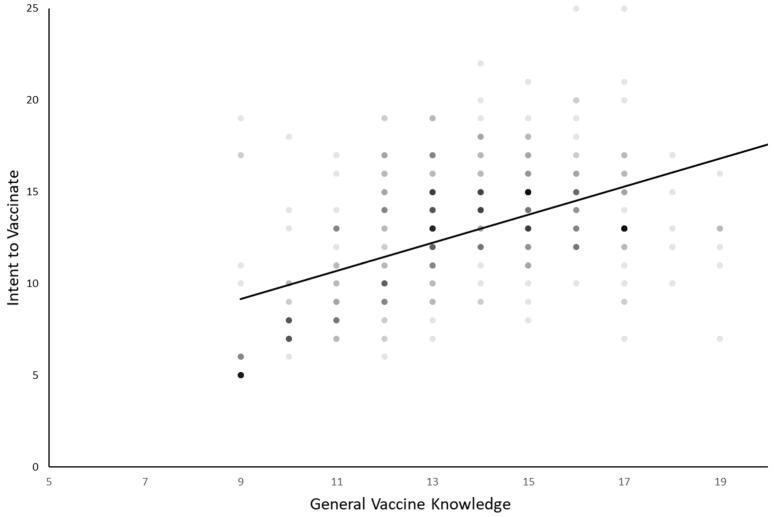
Intent to vaccinate correlates with general vaccine knowledge: There is a strong positive correlation between an individual’s general knowledge about vaccines and their intent to vaccinate their children against human papillomavirus. Larger values on the y-axis indicate a higher intent to vaccinate against HPV, and larger values on the x-axis indicate more general knowledge about vaccines (r = 0.529658, *p* < 0.00001).

Univariate correlation analysis was preformed to determine whether vaccine knowledge impacts respondents’ intent to vaccinate their children against HPV (Figure 5). There is a strong positive correlation between intent to vaccinate and vaccine knowledge (r = 0.5297, *p* < 0.00001). As individuals have increased knowledge about vaccines, in general, their intent to vaccinate their children against HPV increases. Overall intent to vaccinate more than tripled, with median intent to vaccinate rising from 5 to 19 with increasing knowledge of vaccines.

## 4. Discussion

SEM analysis on the first model (Figure 2) shows that income, religious practice, and positive vaccine attitudes are all predictors of intent to vaccinate against HPV. Positive attitudes toward vaccines, with a value of 0.590, is a far stronger predictor of intent to vaccinate than religious practice or income, with values of 0.158 and 0.157, respectively. If an individual has a generally favorable attitude toward vaccination, it follows that they would choose to consider current health guidelines and vaccinate their children against HPV. If an individual feels that vaccines are ineffective or risky, it is unlikely that they would choose to vaccinate their children. Although religious practice has a slight positive impact on intent to vaccinate, it has a negative impact on vaccine attitudes. Positive vaccine attitudes are the strongest predictor of intent, so the negative effect of religious practice on vaccine attitudes decreases intent to vaccinate. A subset of our population was highly religious, highly educated, and had a high intent to vaccinate, which explains the slight positive relationship between religious practice and intent to vaccinate. The relationship between income and intent to vaccinate could be explained by the assumption that individuals with higher income have access to superior healthcare and, therefore, better HPV vaccine access. 

SEM analysis on the second model (Figure 3) shows that knowledge about HPV is a strong predictor of parents’ intent to vaccinate their children against HPV, with a value of 0.784. If parents understand the possible risk presented by HPV infection, it is understandable that they would want to protect their children through vaccination. This interpretation is further supported by univariate factor analysis, which shows that intent to vaccinate is correlated with belief in the safety and efficacy of HPV vaccination (Figure 4). Religious practice is positively related to vaccine knowledge; this relationship could be explained by the highly religious subset of our sample, who are also highly educated. Religious encouragement of premarital abstinence is positively related to HPV knowledge. 

SEM analysis also shows that the belief that religious adherence protects against HPV is a negative predictor of intent to vaccinate, with a value of −0.164. Religious parents may feel that HPV vaccination is unnecessary for their children. Religious parents may also fear that HPV vaccination could increase their child’s sexual activity, which would negatively impact their intent to vaccinate, if they perceive increased sexual activity as a negative outcome. Although the relationship between intent to vaccinate and a belief that religious adherence protects you from HPV was found to be significant, the relationship is not very strong. However, highly religious individuals are more likely to believe that religious adherence and lifestyle protect against HPV than their less-religious peers. The positive influence of religious practice on this belief could indirectly reduce respondents’ intent to vaccinate. This could also suggest stigmatization of those with HPV, as has been seen elsewhere [20]. 

SEM analysis on the first model did not show a significant relationship between vaccine knowledge and intent to vaccinate. Univariate analysis shows intent to vaccinate is correlated with general vaccine knowledge. Although these results may appear to contradict, univariate analysis is sometimes better at illuminating the relationship between latent variables than SEM on a complex model. In a complex model, the relationships between some variables can be masked by the interaction of other variables. Understanding how vaccines provide protection against various diseases and are tested to ensure that they are reasonably safe, could increase confidence in HPV vaccination, thereby increasing parental intent to vaccinate. 

These findings clarify some of the earlier work on religiosity and HPV [12,13,14,15,21] by taking a two-step approach to how religiosity affects vaccination intent. We explored how religiosity impacts other factors that lead to vaccination decision making, specifically in the context of Christianity in America. Many of these connections are likely to be applicable beyond the United States since the variables concerning religiosity and security are not unique to the United States. The results could, therefore, be widely useful wherever religiosity is high and HPV uptake is low. 

### 4.1. Future Directions

This work will be the basis for future work, looking at targeted interventions, geared towards improving vaccine attitudes among highly religious people. Specifically, these will be focused on the idea of vulnerability to infection, due to a religious expectation of abstinence before marriage, as well as on vaccine and HPV knowledge. We will also examine other religious traditions.

### 4.2. Strengths/Limitations

One of the most important strengths of the study is that it was carried out among a targeted group that has a historically low acceptance of the HPV vaccine. Another strength is that our computer models were able to determine a path, where we could examine the effects of variables, such as religious practice or teaching on sexual behaviors, on other variables that influence vaccine decision making. The ideas we examined can potentially be affected by public health interventions. One of the primary limitations to the study is that it was difficult to find people who fit the inclusion criteria who had not finished high school. This suggests a possible bias in the surveyed population towards more educated, wealthier individuals. Given the COVID-19 pandemic, this is a time of potential flux in vaccine attitudes, as governments and individuals incorporate experience with widespread deadly disease, vaccine requirements, and fatigue for government interventions [22]. Continued research will be necessary to ensure that our findings remain consistent.

## 5. Conclusions

The novelty of this work lies primarily in the dissection of the interactions between religious factors and vaccine attitudes. We were able to find not just associations but mechanisms through which religious practice and teachings about sexuality can affect HPV vaccine attitudes. We found that the more knowledge individuals have about HPV and better they understand the risks presented by HPV infection, the higher their intent to vaccinate their children against HPV. We also found that the individuals who viewed vaccines positively were more willing to vaccinate their children against HPV.

In addition, individuals who believe that religious adherence provides protection against HPV have lower intent to vaccinate their children against HPV, which lowers one’s intent to vaccinate. SEM revealed that the sense of safety, knowledge about HPV, and knowledge about vaccines indirectly, rather than directly, influences intent to vaccinate. 

Interventions focused on explaining the risks presented by HPV infection and benefits of vaccination could help increase vaccine acceptance and uptake. Interventions should address general vaccine concerns and highlight testing and safety. Religiosity is associated with the idea that religious beliefs or behaviors will protect a person’s children from infection with HPV. Intervention strategies could, therefore, focus on the dire or fatal consequences of HPV infection, in the event that the children contract the virus, no matter what the circumstances. Other interventions may focus on the high prevalence of the virus and show that people, with which this religious group identifies, are commonly infected with HPV.

## Figures and Tables

**Table 1:** Respondent characteristics.

Range	Number	Percent of Total Responses
Age (*n* = 442)		
18–25	38	8.60%
26–35	170	38.45%
36–45	173	39.14%
46–55	37	8.37%
Over 55	24	5.43%
Gender (*n* = 422)		
Male	175	39.60%
Female	267	60.4%
Non-binary/third gender	0	0%
Prefer not to answer	0	0%
Race/Ethnicity (*n=* 442)		
American Indian/Alaska Native	3	0.67%
Asian	12	2.67%
Black/African American	40	8.91%
Hispanic/Latino	25	5.57%
Native Hawaiian/Pacific Islander	0	0
White	368	81.96%
Prefer not to answer	1	0.22%
Marital status (*n* = 442)		
Single	54	12.22%
Partnered	332	75.11%
Married	19	4.30%
Divorced	7	1.58%
Widow/widower	30	6.79%
Number of Children (*n* = 442)		
One	130	29.41%
Two	220	49.77%
More than Two	92	20.81%
Education (*n* = 442)		
Have not finished high school	12	2.71%
Finished high school	115	26.02%
Some college	79	17.87%
Associate degree	53	12.00%
Bachelor’s degree	84	19.00%
Post-baccalaureate	99	22.40%
Income (*n* = 442)		
Less than $5000	14	3.17%
$5000–$9999	7	1.58%
$10,000–$14,999	14	3.17%
$15,000–$19,999	11	2.49%
$20,000–$29,000	43	9.72%
$30,000–$39,999	36	8.14%
$40,000–$49,999	32	7.24%
$50,000–$59,999	38	8.60%
$60,000–$74,999	39	8.82%
$75,000–$99,999	55	12.42%
$100,000–$124,999	49	11.09%
$125,000–$149,999	48	10.86%
$150,000 or more	56	12.67%
Specific Christian religious affiliation (*n* = 442)		
Anglican/Episcopalian	4	0.90%
Baptist	58	13.12%
Catholic	136	30.77%
Christian (non-denominational)	172	38.91%
Church of Christ/Disciples of Christ	7	1.83%
Congregational	3	0.68%
Jehovah’s Witness	4	0.90%
LDS (Mormon)	3	0.68%
Lutheran	4	0.90%
Methodist/Wesleyan	7	1.58%
Orthodox (Eastern)	4	0.90%
Pentecostal/Charismatic	15	3.39%
Protestant (other)	20	4.52%
Reformed/Presbyterian	2	0.45%
Seventh-day Adventist	1	0.23%
Other	2	0.45%

**Table 2:** Fit statistics for each measurement model.

Model (Latent Variables)	TLI	CFI	RMSEA	SRMR	Chi-Square Test		
					χ^2^	df	*p*-Value
Model A (*religious practice, pro-vaccine religious views, trust in modern medicine, vaccine knowledge, positive attitudes toward vaccines, and intent to vaccinate*)	0.948	0.955	0.041	0.055	5321.84	351	<0.001
Model B (*religious practice, religious encouragement of premarital abstinence, beliefs that religious adherence protects against HPV, HPV knowledge, and intent to vaccinate*)	0.927	0.938	0.058	0.065	4455.42	210	<0.001
Model for the remaining variables (*religious influence, religious hope, parent/peer influence on sexual behavior, fear of HPV vaccine side-effects*)	0.966	0.973	0.041	0.045	2699.05	120	<0.001
Combined model	0.902	0.911	0.042	0.065	13,280.52	1485	<0.001

**Table 3:** Fit statistics for each structural equation model.

Model (Latent Variables)	TLI	CFI	RMSEA	SRMR	Chi-Square Test		
					χ^2^	df	*p*-Value
Model A	0.939	0.946	0.043	0.061	5531.44	378	<0.001
Model B	0.927	0.939	0.058	0.065	4455.42	210	<0.001

## Data Availability

Data may be obtained by contacting the corresponding author.

## Supplementary Materials

The following are available online at https://www.mdpi.com/article/10.3390/vaccines10030397/s1. Table S1: Survey; Figure S1: Confirmatory factor analysis for model 1; Figure S2: Confirmatory factor analysis for model 2.

## References

[1] Brianti P., De Flammineis E., Mercuri S.R. (2017). Review of HPV-related diseases and cancers. New Microbiol..

[2] Braaten K.P., Laufer M.R. (2008). Human Papillomavirus (HPV), HPV-Related Disease, and the HPV Vaccine. Rev. Obs. Gynecol..

[3] McMurray H., Nguyen D., Westbrook T.F., Mcance D.J. (2001). Biology of human papillomaviruses. Int. J. Exp. Pathol..

[4] Phillips A., Patel C., Pillsbury A., Brotherton J., Macartney K. (2017). Safety of Human Papillomavirus Vaccines: An Updated Review. Drug Saf..

[5] Meites E., Szilagyi P.G., Chesson H.W., Unger E.R., Romero J.R., Markowitz L.E. (2019). Human Papillomavirus Vaccination for Adults: Updated Recommendations of the Advisory Committee on Immunization Practices. MMWR Morb. Mortal. Wkly. Rep..

[6] Markowitz L.E., Dunne E.F., Saraiya M., Lawson H.W., Chesson H., Unger E.R., Centers for Disease Control and Prevention (CDC), Advisory Committee on Immunization Practices (ACIP) (2007). Quadrivalent Human Papillomavirus Vaccine: Recommendations of the Advisory Committee on Immunization Practices (ACIP). MMWR Recomm. Rep..

[7] Lin X., Rodgers L., Zhu L., Stokley S., Meites E., Markowitz L.E. (2017). Human papillomavirus vaccination coverage using two-dose or three-dose schedule criteria. Vaccine.

[8] Berenson A.B., Laz T.H., Rahman M. (2016). Reduction in Vaccine-Type Human Papillomavirus Prevalence Among Women in the United States, 2009–2012. J. Infect. Dis..

[9] Canfell K. (2019). Towards the global elimination of cervical cancer. Papillomavirus Res..

[10] Fava J.P., Colleran J., Bignasci F., Cha R., Kilgore P.E. (2017). Adolescent human papillomavirus vaccination in the United States: Opportunities for integrating pharmacies into the immunization neighborhood. Hum. Vaccines Immunother..

[11] Reagan-Steiner S., Yankey D., Jeyarajah J., Elam-Evans L.D., Singleton J.A., Curtis C.R., MacNeil J., Markowitz L.E., Stokley S. (2016). National, Regional, State, and Selected Local Area Vaccination Coverage Among Adolescents Aged 13–17 Years—United States, 2015. MMWR Morb. Mortal. Wkly. Rep..

[12] Chen M.M., Mott N., Clark S.J., Harper D.M., Shuman A.G., Prince M.E.P., Dossett L.A. (2021). HPV Vaccination Among Young Adults in the US. JAMA.

[13] Best A.L., Thompson E.L., Adamu A.M., Logan R., Delva J., Thomas M., Cunningham E., Vamos C., Daley E. (2019). Examining the Influence of Religious and Spiritual Beliefs on HPV Vaccine Uptake Among College Women. J. Relig. Health.

[14] Bernat D.H., Gerend M.A., Chevallier K., Zimmerman M.A., Bauermeister J.A. (2013). Characteristics associated with initiation of the human papillomavirus vaccine among a national sample of male and female young adults. J. Adolesc. Health.

[15] Bodson J., Wilson A., Warner E.L., Kepka D. (2017). Religion and HPV vaccine-related awareness, knowledge, and receipt among insured women aged 18-26 in Utah. PLoS ONE.

[16] Shelton R.C., Snavely A.C., De Jesus M., Othus M.D., Allen J.D. (2013). HPV vaccine decision-making and acceptance: Does religion play a role?. J. Relig. Health.

[17] Thomas T., Blumling A., Delaney A. (2015). The Influence of Religiosity and Spirituality on Rural Parents’ Health Decision Making and Human Papillomavirus Vaccine Choices. Adv. Nurs. Sci..

[18] Kline R.B. (2015). Principles and Practice of Structural Equation Modeling.

[19] MacCallum R.C., Widaman K.F., Zhang S., Hong S. (1999). Sample size in factor analysis. Psychol. Methods.

[20] Ogueji I.A., Adejumo A.O. (2022). Perceived HIV stigmatization and association with cervical screening adoption among HIV-positive women in a Nigerian Secondary Health Facility: Implications for psychological interventions. J. HIV/AIDS Soc. Serv..

[21] Ogueji I.A., Okoloba M.M. (2022). Underlying factors in the willingness to receive and barriers to receiving the COVID-19 vaccine among residents in the UK and Nigeria: A qualitative study. Curr. Psychol..

[22] Gallè F., Sabella E.A., Roma P., Da Molin G., Diella G., Montagna M.T., Ferracuti S., Liguori G., Orsi G.B., Napoli C. (2021). Acceptance of COVID-19 Vaccination in the Elderly: A Cross-Sectional Study in Southern Italy. Vaccines.

