# Supplementary material for: Effects of Religious Practice and Teachings about Sexual Behavior on Intent to Vaccinate against Human Papillomavirus
Source: Vaccines (Basel). 2022 Mar 4;10(3):397. doi: 10.3390/vaccines10030397 (PMC8953177; doi:10.3390/vaccines10030397)
Supplement: Supplementary file 1 [file vaccines-10-00397-s001.zip › Table S1.pdf]

Consent

Implied Consent

Title of the Research Study: Overcoming barriers to HPV vaccine acceptance in religious populations
IRB ID#:

-
My name is Brian Poole, PhD I am a professor at Brigham Young University and I am conducting this research. You are being invited to participate in this research study about vaccination. I am interested to learn more about how you feel about the Human papillomavirus vaccine. Being in this study is optional.
If you choose to be in the study, you will be asked to complete a survey, that should take approximately 20 minutes of your time.
You can skip questions that you do not want to answer or stop the survey at any time. The survey is anonymous, and no one will be able to link your answers back to you. Please do not include your name or other information that could be used to identify you in the survey responses. You will receive your standard compensation from the survey provider for completing the survey.
Questions? Please contact Brian Poole at brian_poole@byu.edu or 801-442-8092. If you have questions or concerns about your rights as a research participant, you can call the BYU Institutional Review Board at 801-422-1461 or irb@byu.edu.
If you want to participate in this study, click the *Accept* button to start the survey.

Accept

Do not accept

Demographic Information

We would like to gather a little bit of demographic data. These questions are helpful to researchers in comparing similar groups of people and to better understand survey results. Although these questions are of a personal nature, the researchers will not be able to connect the information with your name or anything else that could identify you. Please select the best answer for each question.

Religious affiliation:

Buddhism

Christianity

Hinduism

Islam

Judaism

Other

No Religious Affiliation

Do you have a child or children younger than the age of 11?

Yes

No

Number of children:

1

2

More than 2

Age:

Less than 18

18-25

26-35

36-45

46-55

Over 55

Race: Please select all that apply

American Indian or Alaskan Native

Asian

Black or African American

Hispanic or Latino

Native Hawaiian or or Pacific Islander

White

Prefer not to answer

Other (Please specify)

Sex:

Male

Female

Non-binary/third gender

Prefer not to answer

Martial Status:

Single

Partnered

Married

Divorced

Widow/widower

Education:

Have not finished high school

Finished high school

Some college

Associate's Degree

Bachelor's Degree

Post-baccalaureate/professional degree (e.g. Masters, MD, DDS, PhD)

Political Affiliation:

Democrat

Republican

No political affiliation

I prefer not to answer

Other (please describe)

Please indicate your political leanings on economic issues

Very liberal

Liberal

Somewhat liberal

Neither liberal nor conservative

Somewhat conservative

Conservative

Strongly Conservative

Please indicate your political leanings on social issues

Very liberal

Liberal

Somewhat liberal

Neither liberal nor conservative

Somewhat conservative

Conservative

Strongly Conservative

Please indicate your political leanings on scientific issues

Very liberal

Liberal

Somewhat liberal

Neither liberal nor conservative

Somewhat conservative

Conservative

Strongly Conservative

Type of high school attended:

Public

Private/Charter

Homeschooled

Other (please specify)

Type of school your children attend:

Public

Private/Charter

Homeschool

A combination of the above

Other (please specify)

How many people (not including yourself) live in your home?

0

1

2

3

More than 3

How many bedrooms (including guest bedrooms, bedrooms used as offices etc) are in the house or apartment which is your **PRIMARY** residence?

1

2

3

4

More than 4

What is your current employment status? Check **ALL** that apply.

Working full time for pay (Input number of hours below)

Working part time for pay (Input number of hours below)

Not currently employed, looking for work

Retired

Stay at home parent

Disabled (not working because of permanent or temporary disability)

Other (please specify)

Which category best describes your yearly household income before taxes? Include all income received from employment, social security, bank interest, retirement accounts, rental property, investments, etc.

Less then $5,000

$5,000 - $9,999

$10,000 - $14,999

$15,000 - $19,999

$20,000 - $29,999

$30,000 - $39,999

$40,000 - $49,999

$50,000 - $59,999

$60-000 - $74,999

$75,000 - $99,999

$100,000 - $124,999

$125,000 - $149,999

$150,000 or more

Does anyone in your home qualify for government benefits (SNAP, WIC, etc)?

Yes

No

Do you OWN your own home now (includes paying a mortgage)?

Yes

No

Do you OWN a working motor vehicle (car, truck, van, SUV) currently?

Yes

No

Specific Religious Affiliation (Buddhism)

Theravāda (Teaching of the Elders, Southern Buddhism)

Mahāyāna (Great vehicle, East Asian Buddhism)

Vajrayāna (Tantric/Esoteric Buddhism)

Navayāna

Other (please specify)

Specific Religious Affiliation (Christianity)

Anglican/Episcopalian

Baptist

Catholic

Christian (non-denominational)

Church of Christ/Disciples of Christ

Congregational

Jehovah's Witness

LDS (Mormon)

Lutheran

Methodist/Wesleyan

Orthodox (Eastern)

Pentecostal/Charismatic

Protestant (Other)

Reformed/Presbyterian

Seventh-day Adventist

Other (please specify)

Specific Religious Orientation (Hinduism)

Vaishnavism

Shaivism

Shaktism

Smartism

Other (please specify)

Specific Religious Affiliation (Islam)

Shia

Sunni

Sufi

Other (please specify)

Specific Religious Affiliation (Judaism)

Conservative

Orthodox

Reformed

Other (please specify)

Specific Religious Affiliation (Other)

Jainism

Shintoism

Rastafarianism

Sikhism

Confucianism

Zoroastrianism

Pagan/neo-pagan

Traditional African

African Diaspora

Indigenous American

Aboriginal

Folk Religions (please specify)

Other (please specify)

Specific Religious Affiliation (No Religious Affiliation)

Agnostic

Atheist

Spiritual, but no specific affiliation

Other (please specify)

Relationship between HPV and sexual behaviors

Please rate how much you agree with the following statements about Human Papilloma Virus (HPV) and sexual behavior. HPV is transmitted sexually, and can cause certain types of cancer. Vaccination to prevent HPV is often offered for children in the pre-teen years.

	Strongly agree	Somewhat agree	Neither agree nor disagree	Somewhat disagree	Strongly disagree
I do not need to vaccinate my children against HPV because HPV is sexually transmitted, therefore my family's values will protect my children from contracting HPV	○	○	○	○	○
The fear of getting HPV helps prevent premarital sex	○	○	○	○	○
People with diseases caused by HPV are responsible for their own suffering, because the virus is only transmitted through promiscuous sexual practices	○	○	○	○	○
Vaccinating my children against HPV sends them mixed messages about sexual activity	○	○	○	○	○
Vaccinating my children against HPV will make them more likely to have premarital sex	○	○	○	○	○

Outcomes

We would now like to ask you a few questions about the Human Papillomavirus Vaccine. Please rate how much you agree with the following statements about the Human Papillomavirus (HPV) va
Edit Question Label
ccine. HPV is a primarily sexually transmitted virus. Vaccination for HPV is often offered for pre-teens.

	Strongly agree	Somewhat agree	Neither agree or disagree	Somewhat disagree	Strongly disagree
The HPV vaccine has serious side effects	○	○	○	○	○
I am likely to vaccinate my children against HPV OR I have vaccinated my children against HPV	○	○	○	○	○
I am likely to recommend that others vaccinate their children against HPV	○	○	○	○	○
The potential side effects of the HPV vaccine will prevent me from vaccinating my children against HPV	○	○	○	○	○
Since HPV is sexually transmitted, I will not vaccinate my children against it	○	○	○	○	○
I will (or would) vaccinate both my daughters and sons against HPV	○	○	○	○	○
The HPV vaccine would protect my child in the case of sexual assault	○	○	○	○	○

Please rate how much you agree with the following statements about vaccines in general

	Strongly agree	Somewhat agree	Neither agree or disagree	Somewhat disagree	Strongly disagree
Vaccines are more helpful than harmful	○	○	○	○	○
Vaccines often have severe side effects	○	○	○	○	○
Vaccines contain dangerous toxins	○	○	○	○	○
Vaccines are effective at preventing disease	○	○	○	○	○
My children are up to date on their recommended vaccines	○	○	○	○	○

Knowledge about HPV and Vaccines

Now we would like to ask you a few questions about HPV infection and disease. Please rate the following statements about HPV

	Definitely true	Probably true	Neither true nor false	Probably false	Definitely false
HPV is a potentially life-threatening infection	○	○	○	○	○
HPV infection can cause severe physical suffering	○	○	○	○	○
Only a small minority of people will catch HPV during their lives	○	○	○	○	○
HPV causes a substantial amount of cancer	○	○	○	○	○
HPV causes cancer in women but not men	○	○	○	○	○
The HPV vaccine is effective at preventing almost all cancers caused by HPV	○	○	○	○	○

Please rate the truth of the following statements about vaccines in general

	Definitely true	Mostly true	Slightly true	Slightly false	Mostly false	Definitely false
Smallpox has been eliminated because of mass vaccination	○	○	○	○	○	○
Vaccination increase the risk of allergies	○	○	○	○	○	○
Unvaccinated children are more resistant to infections	○	○	○	○	○	○
Routine childhood vaccines can be given to a child taking antibiotics for an ear infection	○	○	○	○	○	○
Current scientific evidence supports associations between vaccines and chronic conditions such as autism or multiple sclerosis	○	○	○	○	○	○
The Food and Drug Administration (FDA) approval process for vaccines is the same as that for other drugs and pharmaceuticals	○	○	○	○	○	○

Religious Practice

These questions will ask about your religious activity and attendance at religious functions. Please answer how often you did these things WITHOUT COVID restrictions: for example before restrictions on attending church in person were in place.

	More then once a day	Once a day	More then once a week	Once a week	More then once a month	Less than once a month
How often do you read scriptures/holy texts?	○	○	○	○	○	○
How often do you attend Sunday School, religious classes or seminars?	○	○	○	○	○	○
How often do you pray?	○	○	○	○	○	○
How often do you attend organized worship services?	○	○	○	○	○	○
How often do you attend other activities sponsored by a religious group?	○	○	○	○	○	○

Please answer the following questions about the influence of your religion on your life

	No influence	Minimal influence	Some influence	Moderate influence	Strong Influence	Extreme Influence
How much influence do your religious beliefs have on what you wear?	○	○	○	○	○	○
How much influence do your religious beliefs have on what you eat and drink?	○	○	○	○	○	○
How much influence do your religious beliefs have on your choices about whom you associate with?	○	○	○	○	○	○
How much influence do your religious beliefs have on what social activities you undertake?	○	○	○	○	○	○
To what extent do your religious beliefs impact the important decisions that you make?	○	○	○	○	○	○

Please rate how much you agree with the following statements about religious hope

	Strongly agree	Somewhat agree	Neither agree nor disagree	Somewhat disagree	Strongly disagree
A positive afterlife/Heaven exists	○	○	○	○	○
It is possible for all humans to live in harmony together	○	○	○	○	○
Miracles are real	○	○	○	○	○
My suffering will be rewarded	○	○	○	○	○
In the future, my children will be able to lead a better life	○	○	○	○	○

Religious views on vaccines

Please indicate how much you agree with the following statement: My religion promotes the use of vaccines.

Strongly agree

Somewhat agree

Neither agree nor disagree

Somewhat disagree

Strongly disagree

Please indicate how much you agree with the following statement: People who share my religion vaccinate their children

Strongly agree

Somewhat agree

Neither agree nor disagree

Somewhat disagree

Strongly disagree

I agree with my religion's teachings about vaccines

Strongly agree

Somewhat agree

Neither agree nor disagree

Somewhat disagree

Strongly disagree

Which do you feel your religion would most agree with:

Everyone should be vaccinated

People should make up their own minds about how and when to vaccinate their children, but vaccines are a good thing

The religion does not teach one way or the other about vaccines

People should make up their own minds about how and when to vaccinate but vaccines are not encouraged

Vaccines should not be used

Religious influence on sexual behavior

How strongly do you agree with the following statement: My religion encourages me not to engage in sexual behaviors unless I am married to the other individual?

Strongly agree

Somewhat agree

Neither agree nor disagree

Somewhat disagree

Strongly disagree

Rate how much you agree with the following statement: "Having sex before marriage is a sin."

Strongly Agree

Somewhat agree

Neither agree nor disagree

Somewhat disagree

Strongly disagree

I do not adhere to the idea of 'sin'

How much emphasis does your religion place on sexual behavior?

One of the most emphasized parts

More emphasized than most other parts

Equally emphasized as other parts

Less emphasized then most other parts

One of the least emphasized parts

Parental/peer influence on sexual behavior

How much did your parents or caretakers emphasize certain rules or cautions about sexual behavior (such as abstinence before marriage or in a committed relationship) in what they taught you?

Excessively

A lot

Something taught but not emphasized

Almost never

Never

My parents did not offer any teachings on sexual behavior

To what extent are sexual relationships outside of marriage discouraged within your social group?

Extremely discouraged

Strongly discouraged

Discouraged

Somewhat discouraged

Rarely discouraged

Not discouraged at all

As a parent, I plan to teach about sexual behavior outside of marriage as follows:

Extremely discouraged

Strongly discouraged

Discouraged

Somewhat discouraged

Rarely discouraged

Not discouraged at all

Trust in modern medicine

We'd like to ask you about your opinions about modern medicine. Please rate how much you agree with the following statements about Modern Medicine

	Strongly agree	Somewhat agree	Neither agree nor disagree	Somewhat disagree	Strongly disagree
Doctors (in general) care about their patients' health just as much or more than their patient's do	○	○	○	○	○
Doctors sometimes do not pay attention to or disregard what their patients are telling them	○	○	○	○	○
Doctors are competent, careful and well trained	○	○	○	○	○
Doctors are totally honest in telling their patients about all of the different treatment options available for their conditions.	○	○	○	○	○
Prescribed treatments are more beneficial than harmful	○	○	○	○	○
A doctor would never mislead you about anything	○	○	○	○	○
Doctors think only about what is best for their patients.	○	○	○	○	○
Doctors always use their very best skill and effort on behalf of their patients.	○	○	○	○	○

Powered by Qualtrics
