# Supplementary figures and images for: Effects of Religious Practice and Teachings about Sexual Behavior on Intent to Vaccinate against Human Papillomavirus
Source: Vaccines (Basel). 2022 Mar 4;10(3):397. doi: 10.3390/vaccines10030397 (PMC8953177; doi:10.3390/vaccines10030397)

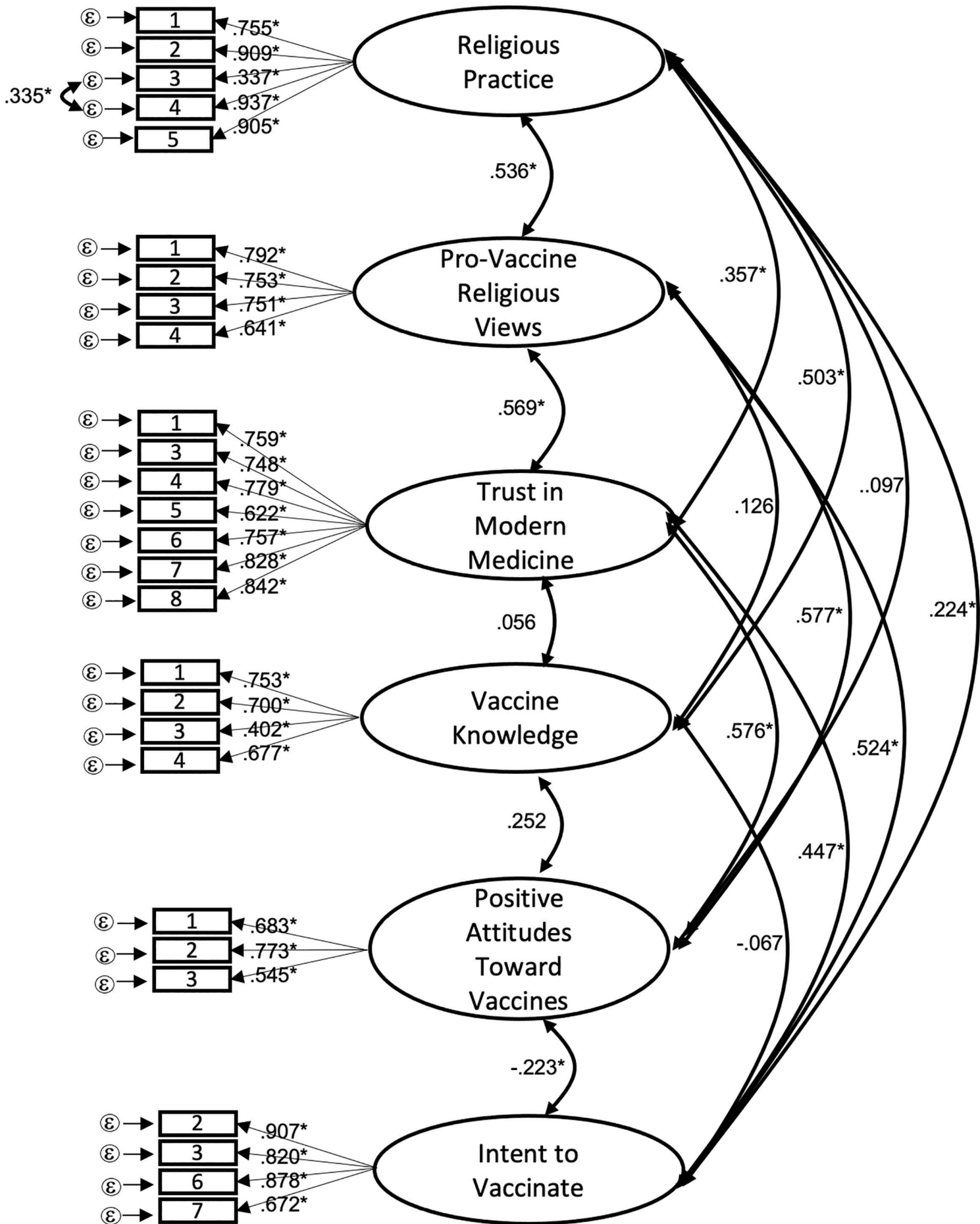

Supplement: Supplementary file 1 [file vaccines-10-00397-s001.zip › Figure S1.pdf]

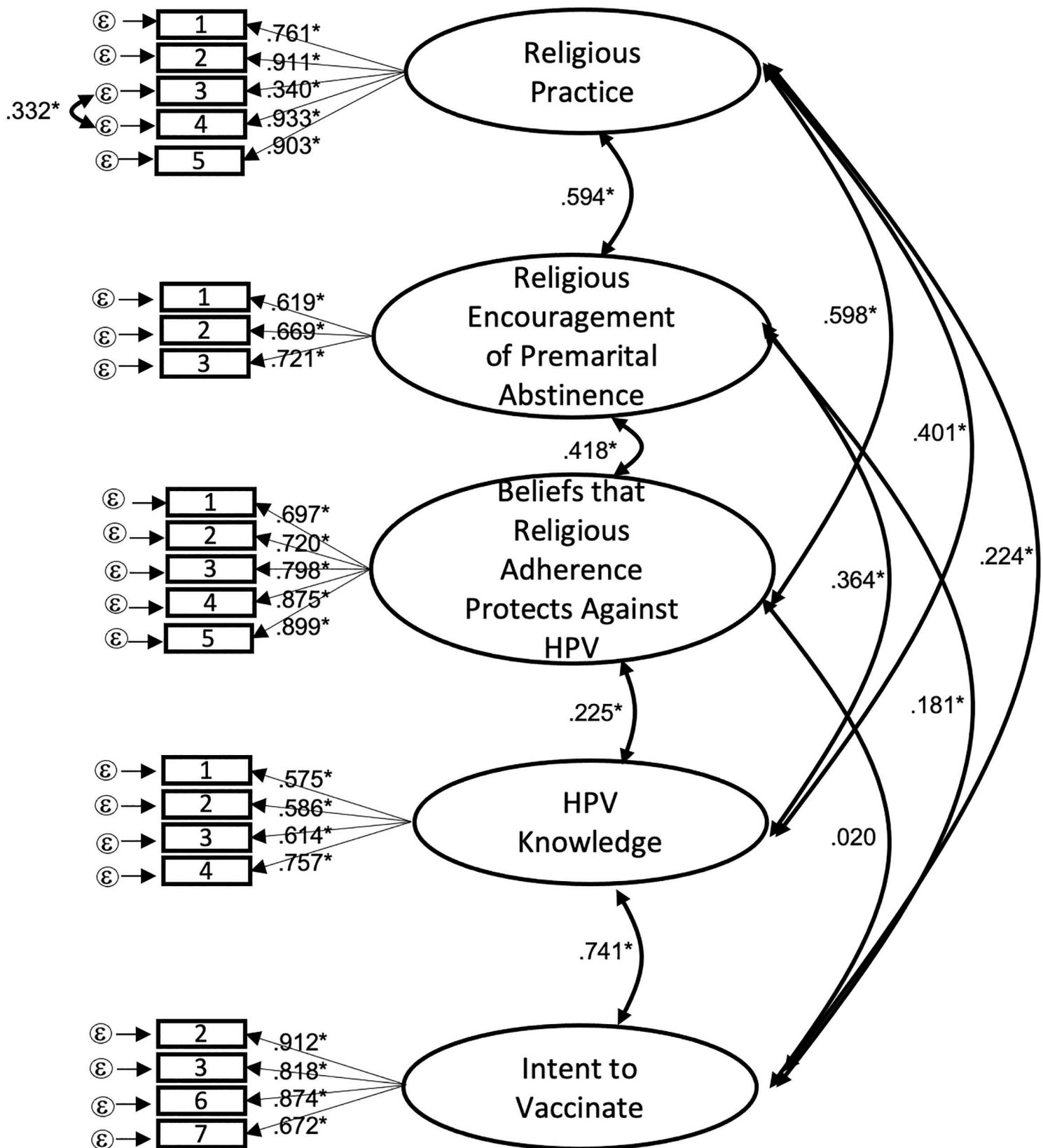

Supplement: Supplementary file 1 [file vaccines-10-00397-s001.zip › Figure S2.pdf]
